# Supplementary material for: Alcohol Prevention in Urgent and Emergency Care (APUEC): Development and Evaluation of Workforce Digital Training on Screening, Brief Intervention, and Referral for Treatment
Source: Int J Environ Res Public Health. 2023 Nov 7;20(22):7028. doi: 10.3390/ijerph20227028 (PMC10671475; doi:10.3390/ijerph20227028)
Supplement: Supplementary file 1 [file ijerph-20-07028-s001.zip › Supplementary File S1.pdf]

Supplementary File S1. Application of the TIDieR checklist to APUEC digital training.

**BRIEF NAME**	
Provide the name or a phrase that describes the intervention.	Alcohol Prevention in Urgent and Emergency Care' (APUEC) is a digital training package for healthcare professionals and healthcare trainees.
**WHY**	
Describe any rationale, theory, or goal of the elements essential to the intervention.	The aim of APUEC is to increase users' knowledge, confidence and skills in screening, brief intervention and referral for treatment (SBIRT) for alcohol prevention in urgent and emergency care settings, through provision of a) education on the impact of alcohol and the role of UECS in alcohol prevention; and b) practical guidance on patient assessment, delivery of brief advice and making referral decisions. Providing employees with access to the APUEC training will increase users' knowledge, skills, and confidence to engage in alcohol Screening, Brief Intervention and Referral for Treatment. This in turn will facilitate the delivery of SBIRT in urgent and emergency care settings.
**WHAT**	
Materials: Describe any physical or informational materials to be used in the intervention, including those provided to participants or used in intervention delivery or in training of intervention providers. Provide information on where the materials can be accessed (e.g., online appendix, URL).	The APUEC training is a reusable learning object (RLO) developed using ASPIRE methodology. RLOs are short, self-contained, multimedia web-based resources including audio, text, images and /or video and which engage the learner in interactive learning towards a single learning objective or goal. This RLO is hosted on HELM Open which is an open-access repository of brief learning resources.<br><br>Content and presentation were developed in collaboration with end-users and provide flexibility for access, inclusivity for people with disabilities, and have low technological skill requirement.<br><br>It can be accessed at https://www.nottingham.ac.uk/helmopen/rlos/practice-learning/public-health/apuec
Procedures: Describe each of the procedures, activities, and/or processes used in the intervention, including any enabling or support activities.	The 'pre-planned schedule' consists of 12 sections which is considered the full 'dose' of intervention content, designed to be accessed in succession. The final section is an activity.
**WHO PROVIDED**	
For each category of intervention provider (e.g., psychologist, nursing assistant), describe their expertise, background and any specific training given.	The content development was led by health psychologists, an emergency medicine doctor, and nurses from an emergency department. The team had expertise in emergency medicine and nursing, psychology, public health, health pro-motion, alcohol prevention, brief interventions, and behaviour change. Content was developed collaboratively through the ASPIRE 6-step process described in the manuscript, (involving 22 contributors, 9 content and technical developers, 10 expert peer reviewers). APUEC is accessed independently by users.
**HOW**	

Describe the modes of delivery (e.g., face-to-face or by some other mechanism, such as internet or telephone) of the intervention and whether it was provided individually or in a group.	The mode of delivery is remote via internet. The APUEC training is accessed individually via a web link, from any PC or mobile device.
**WHERE**	
Describe the type(s) of location(s) where the intervention occurred, including any necessary infrastructure or relevant features.	APUEC can be accessed at any time and in any location, according to user preferences.
**WHEN and HOW MUCH**	
Describe the number of times the intervention is intended to be delivered and over what period of time, including the number of sessions, their schedule, and their duration, intensity or dose.	Although it is recommended that users interact with sections in sequence, users can choose the 'actual schedule' (the order of sections visited or re-visited), the 'dose' they receive (how much of the content they access), the 'duration' (how long they access it for), and the 'intensity' (how often they access it) of the intervention. Repeat visits are possible and encouraged.
**TAILORING**	
If the intervention was planned to be personalised, titrated or adapted, then describe what, why, when, and how.	APUEC is highly flexible to user needs and preferences, meaning that, although the content provided is the same for all users, the *way in which it is used* is highly personalised (as above), and accessibility features enhance the ability to personalise interaction.
**MODIFICATIONS**	
If the intervention was modified during the course of the study, describe the changes (what, why, when, and how).	The intervention was not modified during the study.
**HOW WELL**	
Planned: If intervention adherence or fidelity was assessed, describe how and by whom, and if any strategies were used to maintain or improve fidelity, describe them.	Usage, fidelity, and engagement with APUEC was determined through a self-report evaluation survey and post-exposure qualitative interviews.
