# Supplementary material for: Alcohol Prevention in Urgent and Emergency Care (APUEC): Development and Evaluation of Workforce Digital Training on Screening, Brief Intervention, and Referral for Treatment
Source: Int J Environ Res Public Health. 2023 Nov 7;20(22):7028. doi: 10.3390/ijerph20227028 (PMC10671475; doi:10.3390/ijerph20227028)
Supplement: Supplementary file 1 [file ijerph-20-07028-s001.zip › Supplementary File S2.pdf]

Supplementary File S2. Mapping of design principles to RLO design feature.

**Design Principle**	**Learning Approach for the RLO**
Multimedia	Combination of words, video, and images.
Segmenting	Eleven learner-paced segments (rather than a continuous unit).
Pre-training	Learning outcomes provided, menu provides names of the key concepts.
Modality	Contains animation and narration (in addition to on-screen text).
Coherence	Exclusion of extraneous words, pictures, and sounds through colours, boxes, and moving materials to further resources.
Redundancy	Removal of superfluous on-screen text. Inclusion of slides with visuals and audio only.
Signalling	Use of a menu, navigation buttons, and section numbering.
Spatial contiguity	Ensuring proximity of related words and images.
Temporal contiguity	Ensuring related words and images appear at the same time.
Personalisation	Text is presented in the active voice (conversational style).
Voice	Human narration of text content.

Reproduced (with permission) from: Blake, H.; Fecowycz, A.; Starbuck, H.; Jones, W. COVID-19 Vaccine Education (CoVE) for Health and Care Workers to Facilitate Global Promotion of the COVID-19 Vaccines. *Int. J. Environ. Res. Public Health* **2022**, *19*, 653. https://doi.org/10.3390/ijerph19020653
