# Supplementary material for: Alcohol Prevention in Urgent and Emergency Care (APUEC): Development and Evaluation of Workforce Digital Training on Screening, Brief Intervention, and Referral for Treatment
Source: Int J Environ Res Public Health. 2023 Nov 7;20(22):7028. doi: 10.3390/ijerph20227028 (PMC10671475; doi:10.3390/ijerph20227028)
Supplement: Supplementary file 1 [file ijerph-20-07028-s001.zip › Supplementary File S3.pdf]

Supplementary File S3: Stage 1 Peer Review of Content

Reusable Learning Object Peer Review (1) – Specification

About the Resource

**Title**	Click here to enter text.
**RLO ID**	enter
**Author**	Click here to enter text.
**Email**	Click here to enter text.
**Phone**	Click here to enter text.

About the Review

**Reviewer**	Click here to enter text.
**Date Requested**	Click here to enter text.
**Please complete by**	Click here to enter text.
**Please return to**	Click here to enter text.
**Email**	Click here to enter text.

Your Review

Reviewers, please read through the specification sheet and any additional materials supplied, then complete the 10 questions contained within this form. Wherever possible, please contact the author to discuss your comments and suggest revisions. If necessary, continue on additional sheets and label these with the RLO ID number.

Authors, on receiving the reviewers' comments, please complete the "Author's revision" boxes on the form. You may wish to submit a revised specification and then cross reference the changes onto this form.

Supplementary File S3: Stage 1 Peer Review of Content

1) **Learning Objective**

**Is the learning objective clear and do all sections of the resource support it?**			
Fully ☐	Mostly ☐	Partially ☐	Minimally ☐
**Please outline any revisions you would suggest**			
Click or tap here to enter text.			
**Author's Revisions**			
Click or tap here to enter text.			

2) **Content**

**Is the content factually correct?**			
Fully ☐	Mostly ☐	Partially ☐	Minimally ☐
**Please outline any revisions you would suggest**			
Click or tap here to enter text.			
**Author's Revisions**			
Click or tap here to enter text.			

3) **Text**

**Is the text written in short, clear sentences suitable for narration?**			
Fully ☐	Mostly ☐	Partially ☐	Minimally ☐
**Please outline any revisions you would suggest**			
Click or tap here to enter text.			
**Author's Revisions**			
Click or tap here to enter text.			

4) **Visual Elements**

**Are the suggestions/examples for images/animations/video appropriate?**			
Fully ☐	Mostly ☐	Partially ☐	Minimally ☐
**Please outline any revisions you would suggest**			
Click or tap here to enter text.			
**Author's Revisions**			
Click or tap here to enter text.			
