# Supplementary material for: Alcohol Prevention in Urgent and Emergency Care (APUEC): Development and Evaluation of Workforce Digital Training on Screening, Brief Intervention, and Referral for Treatment
Source: Int J Environ Res Public Health. 2023 Nov 7;20(22):7028. doi: 10.3390/ijerph20227028 (PMC10671475; doi:10.3390/ijerph20227028)
Supplement: Supplementary file 1 [file ijerph-20-07028-s001.zip › Supplementary File S4.pdf]

Supplementary File S4: Stage 2 peer review of media and technical presentation

Reusable Learning Object Peer Review (2) – Media

About the Resource

**Title**	Alcohol Prevention in Urgent and Emergency Care Settings
**URL:**	Click here to enter text.
**RLO ID**	enter
**Author**<br>**Email**	Click here to enter text.<br>Click here to enter text.
**Developer**<br>**Email**	Click here to enter text.<br>Click here to enter text.
**Email**	Click here to enter text.
**Phone**	Click here to enter text.

About the Review

**Reviewer**	Click here to enter text.
**Date Requested**	Click here to enter text.
**Please complete by**	Click here to enter text.
**Please return to**	Click here to enter text.
**Email**	

Your Review

Reviewers: Please work through the RLO at the URL address above. Then complete the relevant sections of this form. You may wish to discuss the feedback with the author. Please indicate below whether this discussion has occurred.

Authors, on receiving the reviewers' comments, please complete the "Author's revision" boxes on the form. You should discuss these with the RLO developer and the mentor before proceeding.

Supplementary File S4: Stage 2 peer review of media and technical presentation

1) **General**

Is the general look and feel of the RLO coherent and conducive for learning?					
Fully	☐	Partially	☐	Minimally	☐
**Please outline any revisions you would suggest**					
Click or tap here to enter text.					
**Author's Revisions**					
Click or tap here to enter text.					

2) **Duration**

Estimate how long would it take students to complete this RLO in your estimation	
Minutes:	Click here to enter text.
**Please outline any revisions you would suggest**	
Click or tap here to enter text.	
**Author's Revisions**	
Click or tap here to enter text.	

3) **Narration**

Is the narration clear, audible and engaging?					
Fully	☐	Partially	☐	Minimally	☐
**Please outline any revisions you would suggest**					
Click or tap here to enter text					
**Author's Revisions**					
Click or tap here to enter text.					

4) **Visual Elements**

Do the images, videos, animations, diagrams, photographs etc support the learning of this subject?					
Fully	☐	Partially	☐	Minimally	☐
**Please outline any revisions you would suggest**					
Click or tap here to enter text.					
**Author's Revisions**					
Click or tap here to enter text.					
