# Supplementary material for: Alcohol Prevention in Urgent and Emergency Care (APUEC): Development and Evaluation of Workforce Digital Training on Screening, Brief Intervention, and Referral for Treatment
Source: Int J Environ Res Public Health. 2023 Nov 7;20(22):7028. doi: 10.3390/ijerph20227028 (PMC10671475; doi:10.3390/ijerph20227028)
Supplement: Supplementary file 1 [file ijerph-20-07028-s001.zip › Supplementary File S5.pdf]

Supplementary File S5. Evaluation survey items

1.To what extent do you agree with the following statements?
Please don't select more than 1 answer(s) per row.

1. I believe patients should be screened for their alcohol consumption in urgent and emergency care settings
   Strongly Disagree
   Disagree
   Neither Agree Nor Disagree
   Agree
   Strongly Agree
2. I believe that urgent and emergency care settings are suitable places to deliver brief interventions for alcohol prevention
   Strongly Disagree
   Disagree
   Neither Agree Nor Disagree
   Agree
   Strongly Agree
3. I believe that brief advice from a healthcare professional can help patients to reduce their drinking and/or seek help with their drinking
   Strongly Disagree
   Disagree
   Neither Agree Nor Disagree
   Agree
   Strongly Agree
4. I believe some patients should be referred for treatment for their alcohol consumption in urgent and emergency care settings
   Strongly Disagree
   Disagree
   Neither Agree Nor Disagree
   Agree
   Strongly Agree
5. I have the knowledge to screen my patients for alcohol consumption
   Strongly Disagree
   Disagree
   Neither Agree Nor Disagree
   Agree
   Strongly Agree
6. I know what tools to use to screen my patients for alcohol consumption
   Strongly Disagree
   Disagree
   Neither Agree Nor Disagree
   Agree
   Strongly Agree
7. I feel confident I can screen my patients for alcohol consumption
   Strongly Disagree
   Disagree

Neither Agree Nor Disagree
Agree
Strongly Agree

8. I have the knowledge to give brief advice to my patients about reducing their alcohol consumption
Strongly Disagree
Disagree
Neither Agree Nor Disagree
Agree
Strongly Agree

9. I feel confident that I can give brief advice to my patients about reducing their alcohol consumption
Strongly Disagree
Disagree
Neither Agree Nor Disagree
Agree
Strongly Agree

10. I have the skills to give brief advice about alcohol with my patients
Strongly Disagree
Disagree
Neither Agree Nor Disagree
Agree
Strongly Agree

11. I intend to increase the number of patients I screen for alcohol consumption
Strongly Disagree
Disagree
Neither Agree Nor Disagree
Agree
Strongly Agree

12. I intend to increase the number of patients I give brief advice to about their alcohol consumption
Strongly Disagree
Disagree
Neither Agree Nor Disagree
Agree
Strongly Agree

2. How helpful has the resource been for learning this subject? *Required*
Very Helpful
Helpful
Unhelpful
Very unhelpful

3. What was your main reason for using this resource? *Required*

4. Were you using this RLO to learn/support learning as a: *Required*
HE student
School student
Tutor/Teacher/Lecturer

Healthcare professional
Patient/Service-user/Carer
Other

5. How did you find out about this resource? *Required*
It was a course learning resource
Recommended by a colleague/peer
Through HELM-Open
Through Open Educational Resource (OER) catalogues
General internet search
Other

6. Did you experience any of these problems in using this resource?
No problems experienced
Technical (please specify)
Level of difficulty/study (please specify)
Language (please specify)
Contextual/cultural differences (please specify)
Other
b. Please specify problem/s experienced:

7. Would you recommend it to others? *Required*
Yes
No

8. What did you most like about this resource? *Required*

9. What did you least like about this resource? *Required*

10. In order to help us track the use of this resource, please let us know which institution or organisation you are from. *Required*

If you would be willing to give more feedback on this resource, please fill in your details below:

The University processes your personal data in order to inform our research on the reuse of open educational resources in healthcare education. The legal basis for this processing is Article 6(1e) of the General Data Protection Regulations "processing is necessary for the performance of a task carried out in the public interest".

Details such as how to contact the University's Data Protection Officer and your rights as a data subject can be found at https://www.nottingham.ac.uk/utilities/privacy/privacy.aspx.

Further details on how your information is processed and how to find out when it will be disposed of can be found at https://www.nottingham.ac.uk/utilities/privacy/privacy-information-for-research-participants.aspx.

11. Name: *Optional*

12. E-mail address: *Optional*
