# Supplementary material for: Alcohol Prevention in Urgent and Emergency Care (APUEC): Development and Evaluation of Workforce Digital Training on Screening, Brief Intervention, and Referral for Treatment
Source: Int J Environ Res Public Health. 2023 Nov 7;20(22):7028. doi: 10.3390/ijerph20227028 (PMC10671475; doi:10.3390/ijerph20227028)
Supplement: Supplementary file 1 [file ijerph-20-07028-s001.zip › Supplementary File S6.pdf]

Supplementary File S6. Interview topic guide

Introduction:

Hi _____________, my name is ______________________Thank you for agreeing to speak with me today about our project. We would like to ask you some questions today about the APUEC package for alcohol prevention in urgent and emergency care. The purpose of this is to evaluate the package with healthcare professionals or trainees. We would like to record this interview, with your permission, so that we can use anonymised quotes from this interview when we produce summaries of our findings. Recordings of the interview will be transcribed in full and anonymised. What you say in the interview is confidential, in that the recordings/transcripts will not be shared with anyone other than the individuals in the research team, although there may be exceptions if you disclose abuse, neglect or potential harm to others which would need to be reported to the appropriate authorities. With your agreement, we may quote some of the things you have said in writing, but these would be anonymous – your name will not appear in any reports.

Before we start, do you have any questions for me?

Are you happy for this interview to be recorded? Thank you.

Questions

Introduction

If we could start with a little background about you – what is your current area of work or study?

Level 1: Reaction

(satisfaction) What is your overall view of this resource?

(engagement) When using the resource, what is your view of the interactive elements? (by this we mean: menu, narration adjustments (can turn on or off and change the speed), video clips, 'i' information boxes, click boxes, quiz, extra resources).

(relevance) How relevant is this resource to you? Or to others? Will you have the opportunity to use this information or apply it in your studies or job?

Level 2: Learning

(Knowledge) Did you learn anything new from using this resource?

(Skill) Did this resource equip you with useful knowledge?

(Attitude) Do you think that alcohol screening, brief intervention and referral is important? (e.g., for individual and societal health?) Have you changed any of your views after using this resource?

(Confidence) Do you feel more confident to deliver alcohol screening, brief intervention and referral (SBIRT) after using this resource? Or talk to people about it?

(Commitment) Are you likely to use any of the information in this APUEC training package in the future? If so, how? Do you intend to share the resource? Tell us more.

Level 3: Behaviour

(Behaviour) Have you applied any of the knowledge from this resource at work (or in your placements/studies)? If so, what and how? Have you done anything differently?

(Required drivers) How do you think this resource should be best used? Who should receive the APUEC resource? How is it best to provide them with access to it? Who should deliver SBIRT? (e.g., which occupational groups) and how can they be supported to do it?

Level 4: Results

(Leading indicators) Have there been any other benefits of using or sharing this resource?

If you have applied this knowledge (e.g., by doing alcohol screening, delivering brief interventions or signposting/referring people to services), do you think it has made any difference? To what degree? (prompt: have you been able to identify people with alcohol problems better? Have you been able to answer people's questions better, give advice better, or signpost people better, or changed anyone's mind about alcohol consumption? Has anyone indicated that they have changed their alcohol habits or accessed services based on your communication with them after you used this resource?

Close: Is there anything else you would like to share with me today?

Thank you for sharing your experiences with me today. It's been really interesting to hear your views.

The package contains further information and resources on alcohol prevention should you feel any need to seek additional support. Package Link: [insert link]

In terms of what happens next, we are speaking with other healthcare professionals and healthcare trainees about their views. We will write up the analysis of these interviews (anonymised) and we intend to publish our project findings in an academic journal.

Thanks again for taking the time to speak with me today.
